# Self-reported anxiety and depression among COVID-19 patients: A prospective cohort study from Turkey: Öykü Turunç

**DOI:** 10.1093/eurpub/ckac129.749

**Published:** 2022-10-25

**Authors:** N Şiyve, AN Emecen, S Keskin, E Başoğlu, Ö Turunç, AF Süner, C Cimilli, B Ünal

**Affiliations:** 1 Department of Public Health, Dokuz Eylul University Faculty of Medicine, Izmir, Turkey; 2 Department of Psychiatry, Dokuz Eylul University Faculty of Medicine, Izmir, Turkey

## Abstract

**Background:**

It has been shown that COVID-19 can cause symptoms and diseases such as insomnia, depression, and anxiety. This study aimed to describe prevalence of feeling anxious or depressive among COVID-19 patients in six months of follow-up time and its association with baseline independent factors.

**Methods:**

This prospective cohort study included patients aged ≥18 years who tested positive for SARS-CoV-2 at Dokuz Eylul University Hospital, Turkey between November 1, 2020 and May 31, 2021. Participants were interviewed by telephone calls on the 1st, 3rd and 6th months after diagnosis. The dependent variable of the study was self-reported moderate or severe anxiety or depression based on EQ-5D-3L general quality of life scale. Generalized estimating equations were used to identify the factors associated with feeling anxious and depressive after SARS-CoV-2 infection.

**Results:**

In total 5446 patients agreed to participate in the study. Frequency of feeling anxious or depressive at the 1st, 3rd and 6th months after diagnosis were 18.5%, 17.9% and 15.4%, respectively. Older age (≥65 years; odds ratio-OR:1.17, 95% confidence interval-CI: 0.95-1.44), female gender (OR:1.76 (1.58-1.96)), bad economic status (OR: 1.62 (1.34-1.97)), having more symptoms (4-5, OR:1.48 (1.21-1.81); ≥5, OR:1.65 (1.35-2.01)), having more underlying health conditions (1-2, OR:1.35 (1.19-1.54); ≥3: OR:1.50 (1.13-1.99)), intensive care unit admission (OR: 2.58 (1.70-3.90)) were associated with self-reported anxiety and depression.

**Conclusions:**

Feelings of anxiety and depression are common in COVID-19 patients and may persist in the long term. Anxiety and depression were associated with gender, economic status and disease severity. Determination of vulnerable groups for anxiety and depression after COVID-19 can be helpful for early diagnosis and initiation of mental care services.

**Key messages:**

• As a consequence of Covid-19, anxiety and depression in Covid-19 survivors are common generally. It shouldn't be overlooked or underestimated for the public mental well-being.

• Covid-19 mental effects on the population have a correlation with social determinants of health. Therefore, determining vulnerable groups is a key to planning mental care services.

